# Function and Expression of Cystic Fibrosis Transmembrane Conductance Regulator after Small Intestinal Transplantation in Mice

**DOI:** 10.1371/journal.pone.0062536

**Published:** 2013-04-23

**Authors:** Penghong Song, Wenfeng Song, Xiaosun Liu, Changhai Jin, Haiyang Xie, Lin Zhou, Biguang Tuo, Shusen Zheng

**Affiliations:** 1 Key Laboratory of Combined Multi-organ Transplantation of Ministry of Public Health, First Affiliated Hospital, School of Medicine, Zhejiang University, Hangzhou, China; 2 Department of Surgery, First Affiliated Hospital, School of Medicine, Zhejiang University, Hangzhou, China; 3 Department of Gastroenterology, Affiliated Hospital of Zunyi Medical College, Zunyi, China; Universidad Europea de Madrid, Spain

## Abstract

The secretion function of intestinal graft is one of the most important factors for successful intestinal transplantation. Cystic fibrosis transmembrane conductance regulator (CFTR) mediates HCO_3_^-^ and Cl^-^ secretions in intestinal epithelial cells. In this study, we made investigation on the expression and function of CFTR in an experimental model of murine small intestinal transplantation. Heterotopic intestinal transplantations were performed in syngeneic mice. The mRNA and protein expressions of CFTR were analyzed by real time PCR and western blot. Murine intestinal mucosal HCO_3_^-^ and Cl^-^ secretions were examined *in vitro* in Ussing chambers by the pH stat and short circuit current (*I_sc_*) techniques. The results showed that forskolin, an activator of CFTR, stimulated jejunal mucosal epithelial HCO_3_^-^ and Cl^-^ secretions in mice, but forskolin-stimulated HCO_3_^-^ and Cl^-^ secretions in donor and recipient jejunal mucosae of mice after heterotopic jejunal transplantation were markedly decreased, compared with controls (*P*<0.001). The mRNA and protein expression levels of CFTR in donor and recipient jejunal mucosae of mice were also markedly lower than those in controls (*P*<0.001), and the mRNA and protein expression levels of tumor necrosis factor α (TNFα) were markedly increased in donor jejunal mucosae of mice (P<0.001), compared with controls. Further experiments showed that TNFα down-regulated the expression of CFTR mRNA in murine jejunal mucosa. In conclusion, after intestinal transplantation, the function of CFTR was impaired, and its mRNA and protein expressions were down-regulated, which may be induced by TNFα.

## Introduction

Intestinal transplantation is currently accepted as a potential therapeutic option for patients with irreversible intestinal failure, including those with short bowel syndrome, who have life-threatening total parental nutrition complications, e.g. total parental nutrition-related liver dysfunction and difficulty of central venous access. In the past 10 years, the outcomes of intestinal transplantation have been improved dramatically, largely resulting from innovative changes in immunosuppression protocols, surgical advances, improved postoperative care, and accumulated experience. However, the intestinal transplantation continues to be one of the more challenging transplants, with a lower survival compared with that of other solid organ transplants, e.g. liver and renal transplantation [1]–[4].

The secretion and absorption of intestine is the most important physiological function of intestine. Therefore, the secretion and absorption function of intestinal graft is one of the most important factors for successful intestinal transplantation. The studies have demonstrated that intestinal absorptive function has been impaired following small intestinal transplantation [5], [6], and the defects in intestinal absorptive function occur even in nonrejecting small intestinal grafts [7], [8]. However, intestinal secretion function after intestinal transplantation is poorly understood. Intestinal secretion not only aids digestion and absorption, but also occurs as a result of some pathophysiologic processes. Intestinal secretion results from the active transports of two principal ions, Cl^-^ and HCO_3_^-^ [9]. Cystic fibrosis transmembrane conductance regulator (CFTR) is a cAMP-dependent Cl^-^ channel with Cl^-^ and HCO_3_^-^ conductance and abundantly expressed in several functionally diverse tissues, such as the pancreas, intestine, kidney, heart, vas deferens, sweat duct and lung [10], [11]. In intestinal epithelial cells, CFTR, which mediates Cl^-^ and HCO_3_^-^ transports, plays an important role in the regulation of intestinal secretion function [9], [12].

In the present study, we made an investigation on the function and expression of CFTR in intestinal graft, which is very important for understanding intestinal physiologic function after intestinal transplantation. The process of intestinal transplantation disturbs intestinal physiological function in various ways, including anatomic changes, ischemia–reperfusion injury, acute and chronic graft rejection, and immunosuppressive therapy. In this study, therefore, we used the mode of syngeneic murine heterotopic jejunal transplantation to seek to identify changes in the function and expression of CFTR in the graft, which is exclusive of the confounding effects of pharmacologic immunosuppression, graft rejection, and graft-versus-host reaction.

## Materials and Methods

### Establishment of Model of Heterotopic Jejunal Transplantation in Mice

Six to ten week male mice with syngeneic C57BL/6 background were used in this study. The mice were purchased from Shanghai Animal Center (Chinese Academy of Science, Shanghai, China) and housed in the experimental animal facility of Zhejiang University under standard care conditions. All animal operative procedure was approved by the Animal Care Committee of Zhejiang University in accordance with the Principles of Laboratory Animal Care (NIH publication 85-23, revised 1985). Murine heterotopic jejunal transplantation was performed according to Zhong et al. [13], with some modifications. Syngeneic C57BL/6 mice were used as donors and recipients. Briefly, after anesthetization by intraperitoneal injection of 50 mg/kg ketamine and 10 mg/kg xylazine, 7 cm donor’s upper portion of jejunum was harvested with the Carrel’s patches of superior mesenteric artery and portal vein which was stored in ice-cold saline until implantation. Using a 10-0 nylon suture, the donor’s Carrel’s patches of superior mesenteric artery and portal vein were anastomosed to the recipient abdominal aorta and inferior vena cava, respectively, yielding end-to-side anastomoses in both cases. The proximal end of the graft was closed with 7-0 silk suture, and the distal end of the graft was exteriorized as a stoma. Recipient mice were sacrificed 2 week after transplantation, and graft and host jejunum were used for experiments.

### Ussing Chamber Experiments

After brief narcosis with 100% CO_2_, the mice were killed by cervical dislocation. The abdomen was opened by midline incision. The jejunal grafts and host jejunums in the experimental mice and jejunums of normal controls were removed and immediately placed in ice-cold iso-osmolar mannitol and indomethacin (1 µmol/L) solution (to suppress trauma-induced prostaglandin release). The jejunums were opened and stripped of external serosal and muscle layers by sharp dissection in the above-mentioned ice-cold iso-osmolar mannitol and indomethacin solution. Ussing chamber experiments were performed as previously described [14]. Briefly, the jejunal mucosae were mounted between two chambers with an exposed area of 0.196 cm^2^ and placed in an Ussing chamber. Parafilm “O” ring was used to minimize edge damage to the tissue where it was secured between the chamber halves. The mucosal side was bathed with unbuffered HCO_3_^–^free modified Ringer’s solution circulated by a gas lift with 100% O_2_ to facilitate the measurement of HCO_3_^-^ secretion by pH stat method. The serosal side was bathed with modified buffered Ringer’s solution (pH 7.4) containing 25 mmol/L HCO_3_^-^ and gassed with 95% O_2_/5% CO_2_. Each bath contained 10.0 ml of the respective solution maintained at 37°C by a heated water jacket. Experiments were performed under continuous short-circuit conditions to maintain the electrical potential difference at zero, except for a brief period (<2 seconds) at each time point when the open-circuit potential difference was measured. Luminal pH was maintained at 7.40 by the continuous infusion of 0.5 mmol.L^−1^ HCl under the automatic control of a pH-stat system (842 Titrando, pH-Stat Controller, Metrohm). The volume of the titrant infused per unit time was used to quantitate HCO_3_^-^ secretion. These measurements were recorded at 5-minute intervals. The rate of luminal HCO_3_^-^ secretion is expressed as µmol.cm^-2^.h^-1^. The rate of Cl^-^ secretion was examined by transepithelial short-circuit current (*I_sc_*; reported as µEq.cm^-2^.h^-1^), which was measured via an automatic voltage clamp (Voltage-Current Clamp, EVC-4000; World Precision Instruments, USA). After a 30-minute measurement of basal parameters, forskolin was added to the serosal side of tissue in Ussing chambers. Changes in jejunal HCO_3_^-^ secretion and *I_sc_* during the 40-minute period ensuing after the addition of forskolin were determined. The mucosal solution used in Ussing chamber experiments contained the following (in mmol/L): Na^+^140, K^+^5.4, Ca^2+^1.2, Mg^2+^1.2, Cl^-^ 120, gluconate 25, and mannitol 10. The serosal solution contained (in mmol/L): Na^+^140, K^+^5.4, Ca^2+^1.2, Mg^2+^1.2, Cl^-^ 120, HCO_3_^-^ 25, HPO_4_^2-^ 2.4, H_2_PO_4_^-^ 2.4, glucose 10, TTX 0.0001 and indomethacin 0.0001. The osmolalities for both solutions were ∼305 mOsm/L.

### RNA Extraction and Real-time RT-PCR

Total RNA from the mucosae of jejunal graft, host jejunum, and control jejunum was extracted using Trizol Reagent (Invitrogen) according to the manufacture’s instruction. The concentrations of all RNA samples were determined spectrophotometrically. The cDNA was produced from 2 µg of total RNA using M-MLV reverse transcriptase (Promega) according to the manufacturer's instructions. Quantitative real-time RT-PCR was performed on a 7500 Fast Real-Time PCR system (Applied Biosystems, USA) using SYBR® Premix Dimer Eraser (Takara Bio, Dalian, China) following the manufacture’s instruction. All samples were run in triplicate and β-actin was used as an internal control. The expression levels of CFTR, solute carrier family 26 member a3 (Slc26a3), solute carrier family 26 member a6 (Slc26a6), tumor necrosis factor α (TNFα), and interferon γ (IFNγ) mRNA were normalized to that of β-actin and were expressed as a ratio relative to β-actin. The primers were as follows: CFTR forward: AAGGCGGCCTATATGAGGTT, reverse: AGGACGATTCCGTTGATGAC; Slc26a3 forward: GAATGCTGATGCAGTTTGCTGAA, reverse: GAGTCCCAGGACAATGGTGAAGA; Slc26a6 forward: TGGTGGTGAAGCTGTTGAATGAC, reverse: ATGTTGCCCACGACATCTACCTC; TNFα forward: AGCCGATGGGTTGTACCTTG, reverse: GACGGCAGAGAGGAGGTTGA; IFNγ forward: CCTGCGGCCTAGCTCTGAG, reverse: GCCATGAGGAAGAGCTGCA; β-actin forward: TACAGCTTCACCACCACAGC, reverse: TCTCCAGGGAGGAAGAGGAT.

### Western Blot Analysis

The mucosae from jejunal graft, host jejunum, and control jejunum were homogenized in lysis buffer (1% Triton X-100, 10 mmol/L Tris, 100 mmol/L NaCl, 1 mmol/L EDTA, 1 mmol/L EGTA, 1% SDS, 0.5% Deoxycholate, 1 mmol/L NaF, 1 mmol/L phenylmethylsulfonyl fluoride, 40 µg of leupeptin/ml, 5 µg of aprotinin/ml, 1 µg of pepstatin/ml) at 4°C. After centrifugation at 14000 rpm for 30 minutes at 4°C, the protein concentrations of supernatants in samples were measured by the BCA protein assay (Pierce, USA). Aliquots of supernatants were used for detecting CFTR, Slc26a3, Slc26a6, TNFα, and INFγ protein using affinity-purified polyclonal antibodies respectively. β-actin was served as internal control. Protein bands were analyzed with image analysis software. Results were expressed as the ratio relative to β-actin.

### Immunohistochemistry Analysis

Immunohistochemical staining was done on the formalin-fixed and paraffin-embedded tissue blocks as previously described [15]. The tissue blocks were cut into 5 µm sections, deparaffinized, and rehydrated. Antigen retrieval was performed in 10 mmol/L citric acid buffer (pH 6.0) in a 750 W microwave for 15 minutes. Endogenous peroxidase activity was blocked with 0.3% hydrogen peroxide in methanol for 15 minutes. After incubation with anti-TNFα antibody (1∶800 dilution, Abcam) overnight at 4°C, the sections were washed in PBS and then incubated with labeled polymer horseradish peroxidase rabbit antibody (Invitrogen) for 1 hour. The sections were rinsed in PBS, incubated with Dako Liquid DAB Large-Volume Substrate-Chromogen System, rinsed gently in distilled water, and counterstained with hematoxylin. Negative controls were also prepared in all assays by replacing anti-TNFα antibody with nonimmune rabbit antiserum. Computer-assisted quantification of the immunostaining was performed as described by Pilette *et al* [16] with an optical microscope (Olympus) equipped with a charge-coupled device (CCD) camera and image analysis software, using a final magnification of 400 ×. Data were collected from an average of 10 randomly selected areas in a random section (average 10 sections) from each examined tissue. Results were expressed as the mean optical density (measured in arbitrary units), representing the mean intensity of staining in the considered area.

### Statistics

All results are expressed as mean ± SEM. ΔHCO_3_^-^ and Δ*I_sc_* both refer to stimulated peak responses minus basal levels. Data were analyzed by one-way analysis of variance (ANOVA) followed by Newman-Keul’s *post-hoc* test or, when appropriate, by the two-tailed student t tests. P<0.05 was considered statistically significant.

## Results

### Expressions of mRNA and Protein of CFTR after Jejunal Transplantation

**Figure 1:**
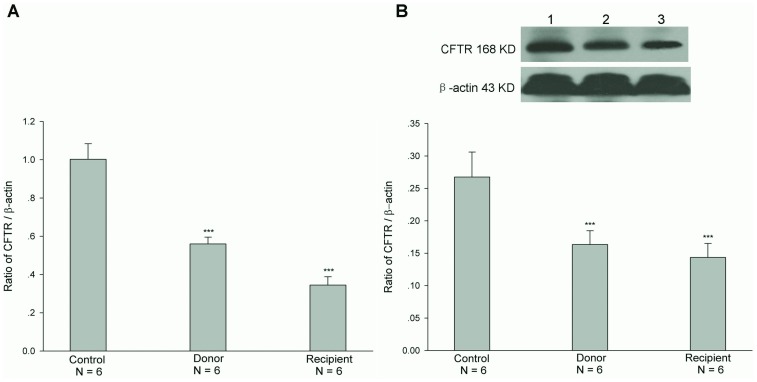
Expressions of mRNA (A) and protein (B) of CFTR in both donor and recipient jejunal mucosae. Upper panel in B is a representative blot graph of CFTR protein. Lane 1: Control; Lane 2: Donor; Lane 3: Recipient. The results are expressed as a ratio to β-actin. Values are mean ± SEM and n = 6 in each series. The mRNA and protein expression levels of CFTR in both donor and recipient jejunal mucosae were markedly lower than those in controls. ***P<0.001.

**Figure 2:**
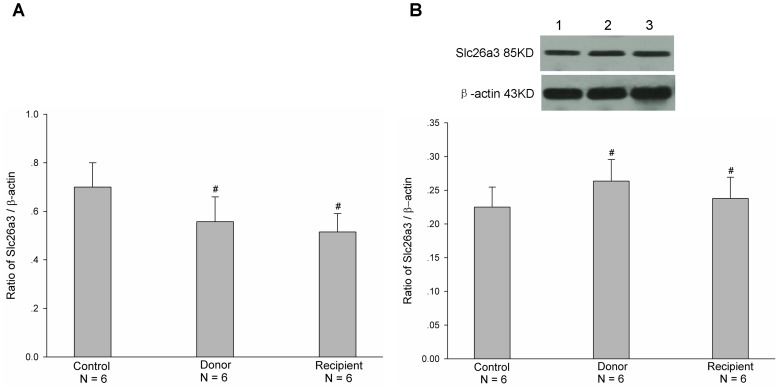
Expressions of mRNA (A) and protein (B) of Slc26a3 in both donor and recipient jejunal mucosae. Upper panel in B is a representative blot graph of Slc26a3 protein. Lane 1: Control; Lane 2: Donor; Lane 3: Recipient. The results are expressed as a ratio to β-actin. Values are mean ± SEM and n = 6 in each series. The mRNA and protein expression levels of Slc26a3 in both donor and recipient jejunal mucosae were not altered significantly, compared with controls.^ #^ *P*>0.05.

**Figure 3:**
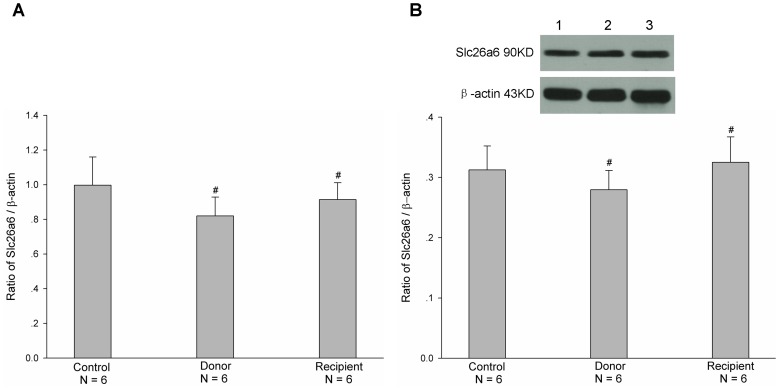
Expressions of mRNA (A) and protein (B) of Slc26a6 in both donor and recipient jejunal mucosae. Upper panel in B is a representative blot graph of Slc26a6 protein. Lane 1: Control; Lane 2: Donor; Lane 3: Recipient. The results are expressed as a ratio to β-actin. Values are mean ± SEM and n = 6 in each series. The mRNA and protein expression levels of Slc26a6 in both donor and recipient jejunal mucosae were not altered significantly, compared with controls.^ #^ *P*>0.05.

Intestinal secretion results from the active transports of two principal ions, Cl^-^ and HCO_3_^-^. It is well known that CFTR, which mediates intestinal epithelial Cl^-^ and HCO_3_^-^ transports, plays an important role in the regulation of intestinal secretion function. Therefore, we firstly examined the mRNA and protein expressions of CFTR in both donor and recipient jejunal mucosae after heterotopic jejunal transplantation. As shown in Figure 1, the mRNA and protein expression levels of CFTR in both donor and recipient jejunal mucosae were decreased after heterotopic jejunal transplantation, compared with controls (P<0.001). In addition to CFTR, Slc26a3 and Slc26a6 are two major Cl^−/^HCO_3_^-^ exchangers and also regulate Cl^-^ and HCO_3_^-^ transports of intestinal epithelial cells [17]. We further examined the mRNA and protein expressions of Slc26a3 and Slc26a6 in donor and recipient jejunal mucosae after heterotopic jejunal transplantation. As shown in Figure 2 and Figure 3, the mRNA and protein expression levels of both Slc26a3 and Slc26a6 in donor and recipient jejunal mucosae were not significantly altered after heterotopic jejunal transplantation, compared with controls (P>0.05). These results indicated that the mRNA and protein expressions of CFTR in intestinal epithelial cells, but not Slc26a3 and Slc26a6, are down-regulated after intestinal transplantation.

### CFTR Function after Jejunal Transplantation

**Figure 4:**
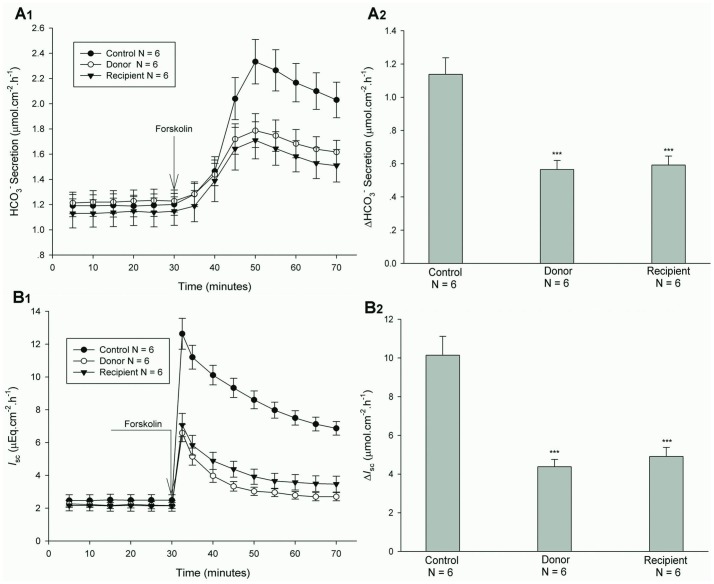
Change of CFTR function after jejunal transplantation in mice. A_1_ represents time course of HCO_3_^–^ secretion and A_2_ represents ΔHCO_3_^–^. B_1_ represents time course of Cl^–^ secretion and B_2_ represents Δ Cl^–^. Forskolin (10 µM) was added at the time indicated by the arrow. Values are mean± SEM and n = 6 in each series. Forskolin-stimulated HCO_3_^–^ and Cl^–^ secretions in both donor and recipient jejunal mucosae were markedly decreased, compared with controls. *** *P*<0.001.

The previous studies have demonstrated that forskolin is a CFTR activator in intestinal mucosal epithelial cells [18], [19]. We examined the alteration of CFTR function after jejunal transplantation through the application of forskolin. As shown in Figure 4, forskolin-stimulated jejunal mucosal epithelial HCO_3_^-^ secretion and *I_sc_* were markedly decreased in both donor and recipient jejunums after heterotopic jejunal transplantation, compared with controls. Forskolin-stimulated jejunal mucosal epithelial net peak HCO_3_^-^ secretion and *I_sc_* were decreased by 50.35% and 56.83% in donor jejunum (P<0.001) and by 47.97% and 51.66% in recipient jejunum (P<0.001), respectively. The results indicated that CFTR function is impaired in both donor and recipient jejunums after heterotopic jejunal transplantation.

### Expressions of mRNA and Protein of Cytokines IFNγ and TNFα after Jejunal Transplantation

**Figure 5:**
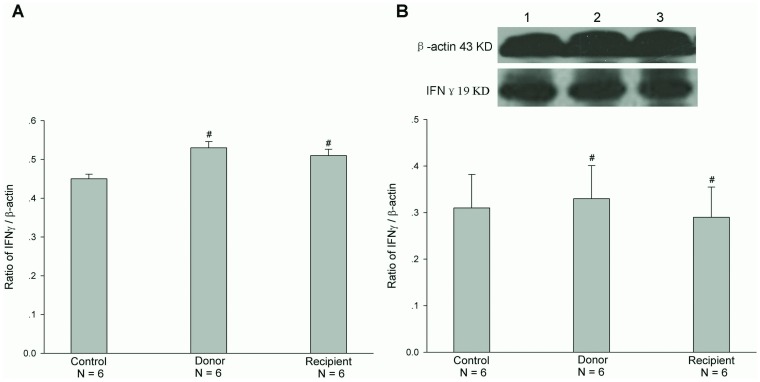
Expressions of mRNA (A) and protein (B) of IFNγ in both donor and recipient jejunal mucosae. Upper panel in B is a representative blot graph of IFNγ protein. Lane 1: Control; Lane 2: Donor; Lane 3: Recipient. The results are expressed as a ratio to β-actin. Values are mean ± SEM and n = 6 in each series. The mRNA and protein expression levels of IFNγ in both donor and recipient jejunal mucosae were not significantly altered, compared with controls. ^#^ *P*>0.05.

**Figure 6:**
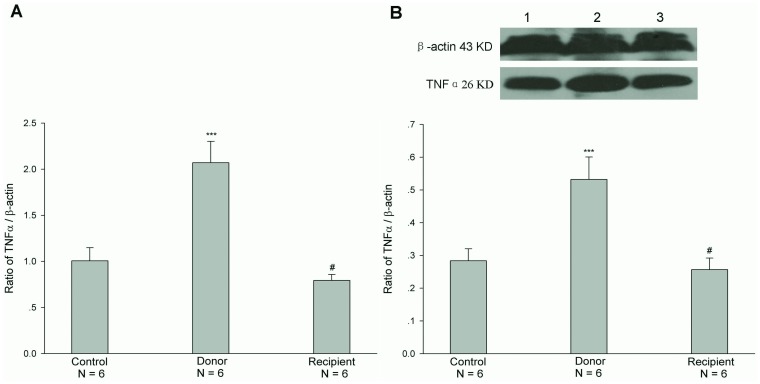
Expressions of mRNA (A) and protein (B) of TNFα in both donor and recipient jejunal mucosae. Upper panel in B is a representative blot graph of TNFα protein. Lane 1: Control; Lane 2: Donor; Lane 3: Recipient. The results are expressed as a ratio to β-actin. Values are mean ± SEM and n = 6 in each series. The mRNA and protein expression levels of TNFα in recipient jejunal mucosa were not significantly altered, compared with controls. However, the mRNA and protein expression levels of TNFα in donor jejunal mucosa were markedly higher than those in controls. ^#^ *P*>0.05; ***P<0.001.

**Figure 7:**
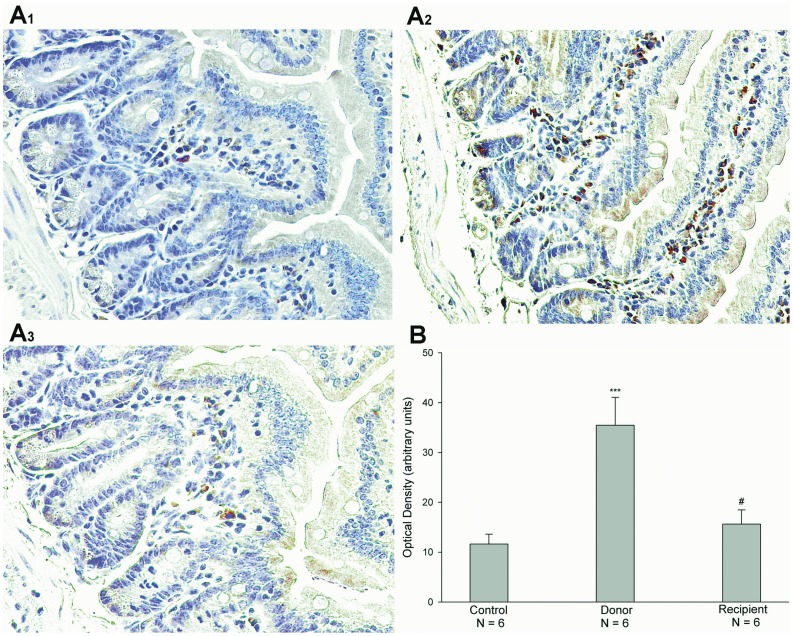
Immunohistochemical analysis of TNFα expression. (A) Representative images for TNFα expressions in the jejunal mucosae of control (A1), donor (A2), and recipient (A3). The brown immunostainings in the cells are TNFα. TNFα is located in the monocytes of intestinal mucosa, and there are not the expressions of TNFα in both villous and cryptal epithelial cells of intestinal mucosa. (B) Expression levels of TNFα in the jejunal mucosae of control, donor, and recipient. The results are expressed as optical density (arbitrary units). Values are mean ± SEM and n = 6 in each series. The expression level of TNFα in the jejunal mucosa of donor was higher markedly than that in control, but there was no significant difference between the expression levels of TNFα in the jejunal mucosae of recipient and control. ***P<0.001, ^#^ *P*>0.05.

The previous studies have shown that cytokines, IFNγ and TNFα, regulated the expression of CFTR in epithelial cells [20]-[22]. Therefore, we examined the mRNA and protein expressions of cytokines IFNγ and TNFα in both donor and recipient jejunal mucosae after heterotopic jejunal transplantation. The results showed that the mRNA and protein expression levels of IFNγ in both donor and recipient jejunal mucosae were not significantly altered after heterotopic jejunal transplantation, compared with controls (P>0.05, Figure 5). The mRNA and protein expression levels of TNFα in recipient jejunal mucosa were not significantly altered either, compared with controls (P>0.05), but the mRNA and protein expression levels of TNFα in donor jejunal mucosa was markedly increased, compared with controls (P<0.001) (Figure 6). We further examined the expression and location of TNFα in jejunal mucosa by immunohistochemistry. The results showed that TNFα was expressed in the monocytes of intestinal mucosa and there were not the expressions of TNFα in both villous and cryptal epithelial cells of intestinal mucosa, and further confirmed the increased expression of TNFα in jejunal mucosa of donor after transplantation compared with control (Figure 7) (P<0.001).

### Effect of TNFα on the mRNA Expression of CFTR in Jejunal Mucosa

**Figure 8:**
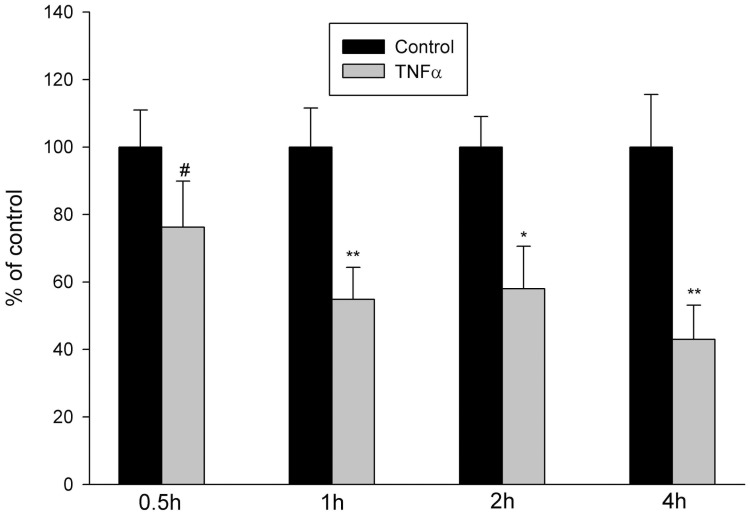
Effect of TNFα on CFTR mRNA expression in jejunal mucosa. The jejunal mucosae in normal mice were treated for different time with TNFα (50 ng/ml) *in vitro*. The RNA extraction and real-time RT-PCR analysis of jejunal mucosae were performed as described in Materials and Methods. The results were expressed as % of control values. Values are mean ± SEM of 6 independent experiments in each series. TNFα markedly increased the mRNA expression level of CFTR in jejunal mocosa after the incubation of 1 hour, but not time-dependent. *P<0.05, **P<0.01.

We further examined the effect of TNFα on the mRNA expression of CFTR in jejunal mucosa through the experiments *in vitro*. As shown in Figure 8, the mRNA expression level of CFTR in jejunal mucosa was markedly decreased at 1 hour after the incubation of murine jejunal mucosa with TNFα, compared with control (P<0.05), but not time-dependent. The results indicated that TNFα may down-regulate the mRNA expression of CFTR in jejunal mucosa.

## Discussion

In the present study, our results demonstrated that after intestinal transplantation, the function of CFTR was impaired, and its mRNA and protein expressions were down-regulated, which may be induced by TNFα.

Although the outcomes of intestinal transplantation have been improved dramatically in the past 10 years, largely through advances in immunosuppression protocols, improved surgical technique and postoperative care, and accumulated experience, the clinical results are not so satisfactory yet, compared with those of other solid organ transplants, e.g. liver and renal transplantation. The intestine functions as both a secretory and an absorptive organ and is in a dynamic state of secretion and absorption, capable of transporting very large volumes of fluid and electrolytes, which is necessary for digestion and physiologic homeostasis. Therefore, the secretion and absorption function of intestinal graft is a factor that possibly contributes to the clinical success of intestinal transplantation. CFTR is a cAMP-activated epithelial Cl^-^ channel with Cl^-^ and HCO_3_^-^ conductance and abundantly expressed in several functionally diverse tissues. In addition to its involvement in epithelial Cl^-^ and HCO_3_^-^ secretions, CFTR also regulates other plasma membrane proteins, including the outwardly rectifying Cl^-^ channels, epithelial Na^+^ channels, K^+^ channels, ATP-release mechanisms, anion exchangers, Na^+^-HCO_3_^-^ cotransporters, and aquaporin water channels [23]-[25]. Thus CFTR might be central in determining transepithelial salt transport, fluid flow, and intracellular ion concentrations. In intestinal epithelial cells, CFTR plays an important role in the regulation of fluid, Cl^-^, and HCO_3_^-^ transports [9], [12]. Intestinal disease in cystic fibrosis is primarily targeted to the small intestine and is characterized by defective alkalinization of secretions in the proximal small intestine, luminal obstruction by thick mucoid secretions, and malabsorption [26]. However, little is known of the function and expression of CFTR after intestinal transplantation. The previous studies have shown that forskolin stimulates intestinal mucosal epithelial HCO_3_^-^ and Cl^-^ secretions and the deletion of CFTR gene completely abolished forskolin-stimulated intestinal mucosal epithelial HCO_3_^-^ and Cl^-^ secretions [18], [19], strongly demonstrating forkolin induces intestinal mucosal epithelial HCO_3_^-^ and Cl^-^ secretions through CFTR. In this study, we found that the mRNA and protein expression levels of CFTR, but not Cl^−/^HCO_3_^-^ exchanges Slc26a3 and Slc26a6, were markedly decreased in both donor and recipient jejunal mucosae after heterotopic jejunal transplantation compared with controls. The further examination found that forskolin-stimulated jejunal mucosal epithelial HCO_3_^-^ and Cl^-^ secretions were markedly decreased in both donor and recipient jejunums compared with controls. The results indicated that the CFTR function of intestinal graft is impaired and its mRNA and protein expressions are down-regulated after intestinal transplantation.

What is responsible for the alterations of function and expression of CFTR after intestinal transplantation? The previous studies have shown that TNFα down-regulated CFTR mRNA expression in HT-29 cells, a colon epithelium-derived tumor cell line, in a dose- and time-dependent fashion [22]. IFNγ, but not IFNα or IFNβ, down-regulated CFTR mRNA levels in two colon-derived epithelial cell lines, HT-29 and T84 cells, in a time- and concentration-dependent manner [20]. And TNFα and IFNγ synergistically decreased CFTR mRNA expressions in HT-29 and T84 cells [27]. In addition, TNFα and IFNγ decreased agonist-stimulated CFTR-mediated Cl^-^ secretion in T84 cells [27], [28]. In this study, therefore, we first examined the mRNA and protein expressions of TNFα and IFNγ in donor and recipient jejunums after heterotopic jejunal transplantation. The results showed that the mRNA and protein expression levels of IFNγ in donor and recipient jejunums were not altered significantly compared with controls, and the mRNA and protein expression levels of TNFα in recipient jejunum was not altered significantly either. However, the mRNA and protein expression levels of TNFα in donor jejunum were markedly higher than those in controls. Immunohistochemical results also showed an enhanced expression of TNFα in donor jejunum. The results indicated that it is possible that TNFα induces the alterations of function and expression of CFTR. Our results showed that the alterations of CFTR expression and function occurred in both donor and recipient jejunums, but the alteration of TNFα expression only occurred in the donor jejunum, indicating that TNFα might exert its effect on the recipient jejunum through blood circulation in addition to its local action on the donor jejunum. The further experiments showed that the incubation of jejunal mucosa with TNFα in vitro decreased the mRNA expression level of CFTR in jejunal mucosa. Taken together, these results indicate that TNFα induces the alterations of function and expression of intestinal CFTR after intestinal transplantation.

TNFα is a potent pro-inflammatory cytokine that regulates essential biological functions (e.g., cell differentiation, proliferation, survival, apoptosis) and a broad spectrum of responses to stress and injury, and plays a critical role in the pathogenesis of chronic inflammatory diseases [29], [30]. It is primarily produced by immune cells such as monocytes and macrophages, but it can also be released by many other cell types, including acinar cells. The studies in the experiments *in vitro* showed that human colonic epithelial cells might produce a wide range of proinflammatory cytokines, including TNF-α, in response to invasive microbial pathogens [31.32]. In this study, our immunohistochemical results showed that TNFα was located in the monocytes of intestinal mucosa, and there were not the expressions of TNFα in both villous and cryptal epithelial cells of intestinal mucosa. The results indicate that TNFα is primarily produced by the monocytes of intestinal mucosa after the intestinal transplantation.

What induced the increase of TNFα in the mucosa of the intestinal graft? In this study, we used the mode of syngeneic murine heterotopic jejunal transplantation, which is exclusive of the effects of graft rejection and graft-versus-host reaction. In addition, the expression of TNFα was only enhanced in the donor jejunal mucosa, not in the recipient jejunal mucosa, after the heterotopic jejunal transplantation, indicating that it is possible that anatomic changes, including transection of the intestinal wall along with intrinsic neurons, complete extrinsic autonomic denervation, and the disruption of lymphatic drainage, or ischemia–reperfusion injury induced the increase of TNFα in the mucosa of the intestinal graft. Although infection and endotoxemia are potent stimulants of TNFα production, the studies also found that intestinal ischemia–reperfusion increased TNFα levels in the serum and intestinal tissue of rat [33]-[35]. In addition, enteric nervous system also plays an important role in the regulation of intestinal immune function. The interactions between the enteric nervous system and local immunocytes are responsible for many functional changes, including motility and secretion. Several neuropeptides, such as tachykinins, vasoactive intestinal peptide, somatostatin, and opioids, are involved in both intrinsic and extrinsic innervation but can also affect the release of cytokines and proinflammatory mediators. On the other hand, proinflammatory mediators, such as eicosanoids and cytokines, may activate intrinsic neurons directly or stimulate extrinsic neurons indirectly, releasing neuropeptides which act on intrinsic neurons, smooth muscle cells, or enterocytes [36], [37]. In several models of experimental inflammation, intrinsic denervation as well as destruction of sensory C fibres affected the local immune reactions. The functional ablation of sensory neurons by capsaicin pretreatment worsened colitis in rabbit [38]. The vagotomy significantly increased the TNF-α levels in serum and colonic tissue in mice [39], [40]. These studies indicate that extrinsic and intrinsic denervations might affect the release of TNF-α in the monocytes of intestinal mucosa.

In conclusion, our study demonstrates that intestinal transplantation impairs the CFTR function and down-regulates the expression of CFTR in intestinal mucosal epithelial cells, even in non-rejecting small intestinal graft. It also implicates that the examination of CFTR mRNA in biopsy specimens following intestinal transplantation may provide useful information for evaluating intestinal graft function.
